# Neuronal adaptation involves rapid expansion of the action potential initiation site

**DOI:** 10.1038/ncomms4817

**Published:** 2014-05-23

**Authors:** Ricardo S. Scott, Christian Henneberger, Ragunathan Padmashri, Stefanie Anders, Thomas P. Jensen, Dmitri A. Rusakov

**Affiliations:** 1 UCL Institute of Neurology, University College London, Queen Square, WC1N 3BG London, UK; 2 Instituto de Neurociencias de Alicante, Universidad Miguel Hernández—CSIC, Campus de San Juan, Sant Joan d’Alacant, 03550 Alicante, Spain; 3 Institute of Cellular Neurosciences, University of Bonn Medical School, D-53105 Bonn, Germany; 4 These authors contributed equally to this work; 5 Present address: University of Nebraska Medical Center, Omaha, Nebraska 68198, USA

## Abstract

Action potential (AP) generation is the key to information-processing in the brain. Although APs are normally initiated in the axonal initial segment, developmental adaptation or prolonged network activity may alter the initiation site geometry thus affecting cell excitability. Here we find that hippocampal dentate granule cells adapt their spiking threshold to the kinetics of the ongoing dendrosomatic excitatory input by expanding the AP-initiation area away from the soma while also decelerating local axonal spikes. Dual-patch soma–axon recordings combined with axonal Na^+^ and Ca^2+^ imaging and biophysical modelling show that the underlying mechanism involves distance-dependent inactivation of axonal Na^+^ channels due to somatic depolarization propagating into the axon. Thus, the ensuing changes in the AP-initiation zone and local AP propagation could provide activity-dependent control of cell excitability and spiking on a relatively rapid timescale.

## Abstract (web summary)

Neuronal adaptation to repetitive stimuli is required for the correct functioning of neuronal networks. Here, the authors show that rapid expansion of the axonal spike-initiation site accompanied by local spike deceleration is the cell adaptation mechanism that responds to repetitive excitatory inputs.

## Graphical abstract

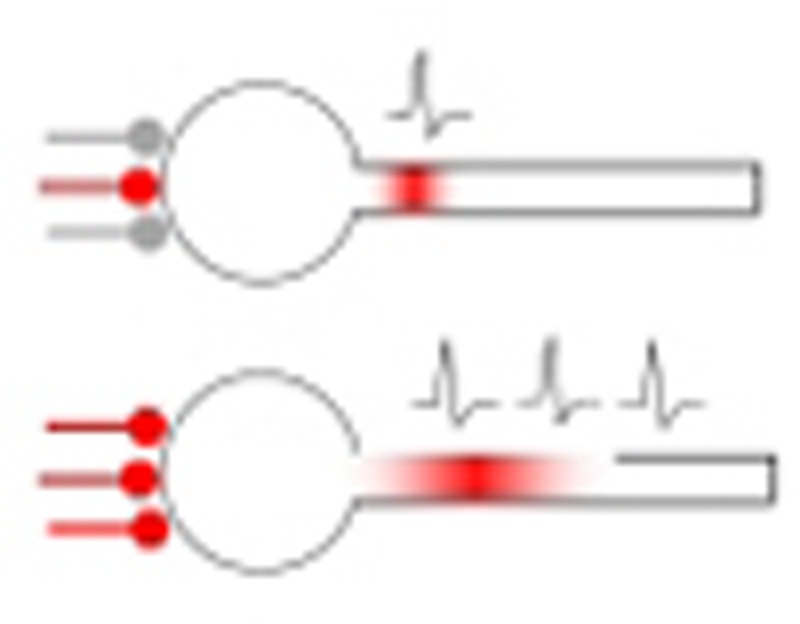

Integration of synaptic inputs leading to initiation and propagation of action potentials (APs) provides the basic machinery of information encoding and transfer in neural circuits. The spike generation threshold, however, could vary depending on the history and dynamics of the cell membrane potential, *in vitro* and *in vivo*^1^,^2^,^3^,^4^,^5^,^6^. In most cases, the cell AP threshold is inversely correlated with the rate of dendrosomatic depolarization, thus providing a clear informational advantage for synchronized input, or for successful coincidence detection, in the microcircuits of the brain^7^,^8^,^9^. However, the cellular machinery underlying this basic adaptation mechanism, including the contributions of somatic and axonal spike initiation, remains under debate.

In most neuronal types, APs are initiated within the axon initial segment, or the first node of Ranvier in myelinated axons, which is normally associated with a hotspot of Na^+^ channels^10^,^11^,^12^,^13^,^14^, although there have been observations suggesting otherwise^15^,^16^. Intriguingly, recent studies have shown that the axonal AP-initiation zone could be modified, on the timescale of hours or days, by sustained network activity^17^,^18^. Such modification has a strong adaptive significance because the position and geometry of the AP-initiation zone reflect the ability of the host neuron to recognize specific spiking patterns, and thus to filter or encode specific network information^19^. At the same time, high-sensitivity voltage imaging in myelinated axons of cortical neurons has indicated stability of the spike-initiation site over shorter timescales^20^, and little threshold variability has been attributed to the axonal spike-initiation mechanism in the cortex^6^. Nonetheless, a very recent study in cortical cells, which combined dual-patch soma–axon recordings with axonal Na^+^ imaging, predicted that minor changes in the Na^+^ channel distribution in the proximal axon could have a strong effect on neuronal excitability^16^. This basic phenomenon was not intuitively understood in the past and may have fundamental implications of our understanding of cell excitability^21^.

To understand how short-term plasticity influences cell excitability, here we focus on spike generation in non-myelinated axons of hippocampal dentate granule cells (mossy fibres, MFs), which represent a major excitatory pathway into area CA3 and exhibit extraordinary use- and target cell-dependent plasticity^22^,^23^,^24^,^25^. Previous MF studies, which utilized presynaptic Ca^2+^ imaging^26^, whole-terminal configuration^27^ and soma–axon dual-patch recording^25^, have indicated significant electrotonic propagation of somatic voltage into these axons. These observations indirectly suggested that excitatory input may influence channel mechanisms of axonal spike initiation in the proximal axon in a distance-dependent manner. This basic biophysical phenomenon has not been reported previously, even though depolarization-dependent inactivation of axonal Na^+^ and K^+^ channels in MFs has been documented in detail using whole-terminal or axonal outside-out patch recordings^28^,^29^. Here we report that reducing the pace of excitatory synaptic input to granule cell dendrites does elevate the spiking threshold of the cell. We use dual-patch soma–axon recordings and axonal Na^+^ and Ca^2+^ imaging to conclude that the underlying mechanism relies on depolarization-dependent partial inactivation of axonal Na^+^ channels, which weakens with greater distances from the soma. This inactivation leads to the site of AP initiation being expanded, spreading away from the soma and decelerating local spikes. Our experimental observations appear consistent with predictions of a detailed biophysical NEURON model of a reconstructed granule cell and Monte Carlo tests. We conclude that rapid use-dependent changes in the spread of the axonal area where APs can be initiated provide the mechanism by which a principal neuron can adapt its information-handling properties to the ongoing excitatory activity.

## Results

### Dendritic excitation controls the granule cell-spiking threshold

**Figure 1:**
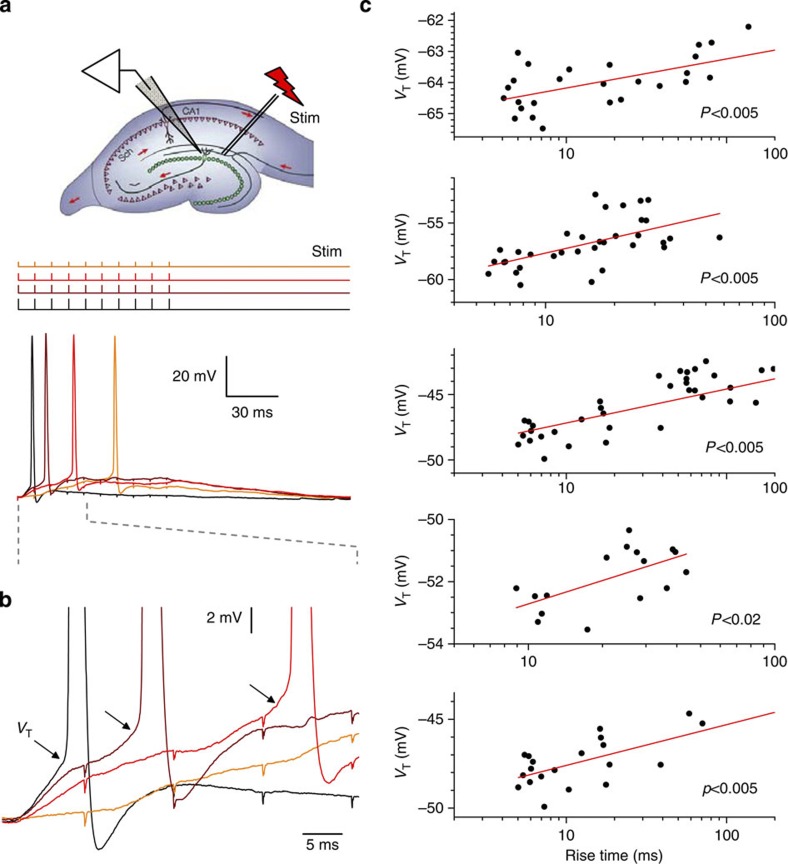
Synaptic input modulates the spiking threshold of dentate granule cells. (**a**) Inset: experiment diagram. Upper traces: pattern of electric stimuli applied with varied amplitudes to the presynaptic perforant fibres, as depicted. Lower traces: the corresponding somatic voltage recordings (colour-coded accordingly) show a characteristic one-cell example. (**b**) Somatic voltage traces in **a** expanded as shown by dashed lines; arrows depict spiking threshold values (*V*_T_) that visibly increase with lower rates of perforant fibre excitation-induced somatic depolarization. (**c**) Multiple trials recorded, in an arbitrary sequence, in five individual cells show strong correlation between the rate of depolarization (set by the synaptic input strength) and the granule cell-spiking threshold (*V*_T_). Experiments carried out at 33 °C.

We held granule cells in whole-cell mode (current clamp) and stimulated perforant path fibres with an extracellular electrode, >200 μm away from the recorded cell, using a 100-Hz train of 10 stimuli (Fig. 1a; temperature ~\n33 °C; GABA_A_ blocked). Stimulation-induced synaptic discharges led to somatic depolarization that was sufficient for the cell to fire, and we varied the stimulus strength from trial to trial so that the rise time of somatic depolarization before the first AP ranged from 5–8 to 100–200 ms. This type of granule cell activity is well within the range documented in behaving animals^30^. We found that in every tested cell, the spiking threshold increased robustly when the depolarization rate was slowed down (Fig. 1b,c). This was not due to homoeostatic changes or use-dependent plasticity in the circuitry because trials with slower and faster depolarization were recorded in an arbitrarily sequence. The maximum increase in the spiking threshold due to deceleration of excitation was 5–8 mV across the sample (Fig. 1). As these cells required a depolarization of 15–30 mV above the resting potential (approximately −75 mV) to generate an AP, our data indicated that accelerating the excitatory input had an effect roughly equivalent to the additional 20–30% input (summated at a slower rate) to initiate cell firing.

### AP-initiation site expands along the axon during spiking

**Figure 2:**
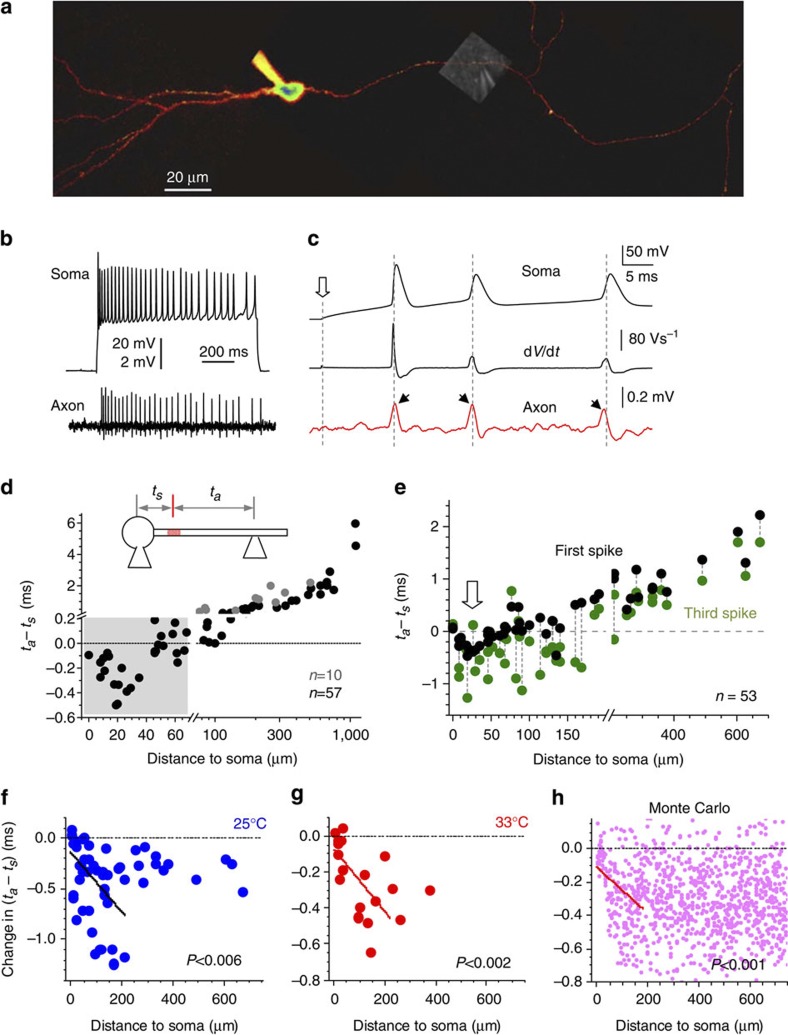
The axonal spike-initiation area broadens away from the soma during somatic excitation. (**a**) Typical experiment: granule cell held in whole-cell mode (40 μM Alexa Fluor 594, *λ*_x_^2*P*^*=*800 nm), with an axonal bouton loose-patched (DIC inset overlay). (**b**) Characteristic spikes at the soma (upper trace) and in the axon (lower trace, loose patch; voltage scales apply accordingly) upon a depolarizing pulse; current clamp. (**c**) Example as in **b** on a magnified timescale; traces, somatic *V*_m_ including three spikes (top), d*V/*d*t* (middle), and axonal spikes recorded at ~\n100 μm from the soma (bottom). Dotted lines, somatic spike onset (d*V/*d*t* peak); arrows, axonal spike peaks (indicating a shift towards an earlier onset for the third axonal spike relative to somatic AP). (**d**) The (*t*_a_*−t*_s_) latency values for somatic (*t*_s_) and axonal (*t*_a_) spikes versus pipette position in the axon (inset). Black and grey dots, data recorded in individual axons from the main branch (*n=*57pairs) and collaterals (*n=*10), respectively. Grey shade, proximal axonal segment on an expanded scale; the AP-initiation site is near the minimal (*t*_a_*−t*_s_) value (20–25 μm from the soma). (**e**) (*t*_a_*−t*_s_) values for the first (black) and the third (green) spikes invoked by somatic depolarization as in **b**; spikes from the same train are connected by dotted lines (*n=*53 soma–axon pairs). Arrows, the approximate position of the AP-initiation site in baseline conditions (first spike). (**f**,**g**) The (*t*_a_*−t*_s_) latency change by the third spike (at 25 and 33 °C, as indicated) is negatively correlated with the axonal distance in the proximal (0–200 μm) segment; lines, linear regression; *n=*53 cells at 25 °C, and *n*=17 at 33 °C. (**h**) Monte Carlo simulations (example) mimicking an expansion of the AP-initiation site by the third spike predicting negative correlation between the (*t*_a_*−t*_s_) latency change and the axonal distance, within the proximal segment as in (**f**,**g**); line, linear regression (*P*<0.001); 1,000 points simulated, see Supplementary Fig. 2 for detail.

The AP axon initiation site was reported to be within ~\n20 μm from the granule cell soma in rats (whole-cell recordings from axonal blebs)^31^ and in mice (loose-patch axonal recordings)^32^. To determine its location in our experiments, we carried out dual-patch recordings from the soma (whole-cell) and the axon (loose patch) of granule cells (Fig. 2a), as detailed previously^25^. In these settings, somatic and axonal events could be readily identified and analysed (Fig. 2b,c). The AP-initiation site was determined by documenting the time lag, or latency (*t*_a_−*t*_s_) between the axonal spike (latency *t*_a_) recorded at different distances from the soma, and the somatic spike (latency *t*_s_; Fig. 2b–d; Methods). We found that the (*t*_a_−*t*_s_) value reached its minimum when the axonal electrode was 20–25 μm from the soma (Fig. 2d), thus indicating the AP-initiation site^33^ consistent with previous observations^31^,^32^. We also found that spikes could propagate along MFs with high fidelity (>99.5% at the highest possible spiking frequency due to somatic depolarization), although a moderate increase in extracellular K^+^ (to 5 mM, compatible with conditions of epileptiform activity) could prompt significant propagation failures (Supplementary Fig. 1).

Under baseline conditions, we also compared (*t*_a_−*t*_s_) values for the first and the subsequent spikes during 200-ms depolarization. By the third consecutive AP, the minimal (*t*_a_−*t*_s_) values became spread over a 100- to 120-μm-long axonal segment, in sharp contrast with the highly localized site for the first spike (Fig. 2e, black and green dots). This spread was quantitatively reflected in the negative regression, within the proximal axonal segment (0–200 μm), between the use-dependent change in (*t*_a_−*t*_s_) and the distance from the soma (*n*=43, *P*<0.006 at 25 °C, and *n*=17, *P*<0.002 at 33 °C; Student’s *t*-test for regression slope; Fig. 2f,g); at the same time, (*t*_a_−*t*_s_) values remained negative over the longer distances (at *P*<0.001 for distance >250 μm, one-sample *t*-test). The initial negative slope along with the negative value of distant data in the scatter were consistent with the spike-initiation site expansion predicted by straightforward Monte Carlo tests of (Fig. 2h, Supplementary Fig. 2a–c). However, the fact that (*t*_a_−*t*_s_) values partly returned closer to zero values at >200 μm also suggested a contribution of activity-dependent spike deceleration in proximal segments^32^ (Supplementary Fig. 2d–f). The overall effect appeared to reach its peak by the third spike (30–50 ms after the depolarization onset, Supplementary Fig. 3).

Importantly, by the third spike the average variability of the (*t*_a_−*t*_s_) values among cells increased considerably (compare the vertical scatter of black and green dots in Fig. 2e). This was a direct consequence of increased trial-to-trial (*t*_a_−*t*_s_) variability in individual axons: the s.d. of measured (*t*_a_−*t*_s_) values increased from 60±10 μs for the first spike to 180±35 μs for the third spike at 25 °C (measured in *n*=14 cells, *P*<0.006; *t*-test here and thereafter) and from 47±13 to 82±19 μs at 33 °C (*n*=9, *P*<0.005). Such two- to threefold increases in the variability could not be explained by an increased latency measurement error by the third spike (its amplitude decreased by only 20–30%) but most likely reflected the uncertainty, or the stochastic nature, of the AP-initiation point along an expanded segment of the proximal axon. Clearly, the increased variability of (*t*_a_−*t*_s_) values could also be partly due to use-dependent deceleration of local spikes. In summary, these data suggest that somatic excitation resulted in the probability of spike initiation distributed more evenly along the proximal axonal segment, compared with the resting conditions.

### AP-initiation site expands during subthreshold excitation

**Figure 3:**
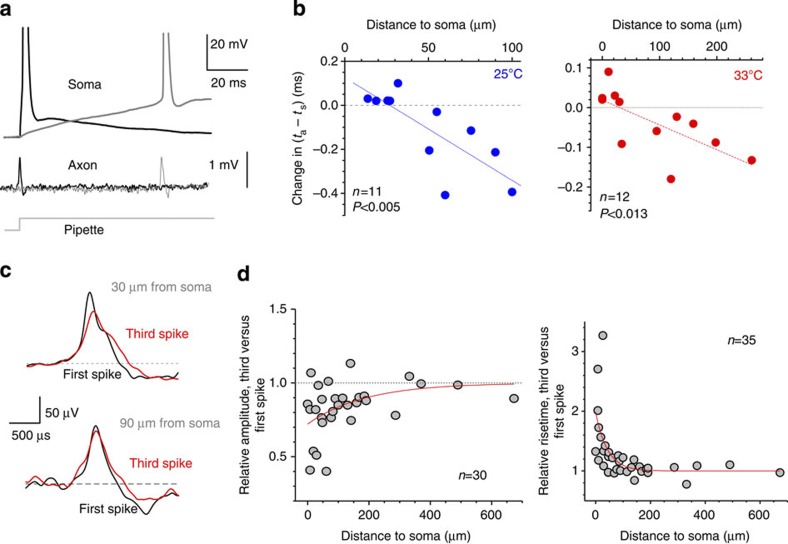
Depolarization broadens the spike-initiation area slowing down local spikes. (**a**) Characteristic recordings of somatic (upper trace) and axonal (lower trace, ~\n75 μm from the soma) spikes generated following shorter (black) and longer (grey) subthreshold depolarization periods, as indicated. (**b**) A summary of changes in the (*t*_a_*−t*_s_) latency plotted for different recording positions in the axon (compare with Fig. 2f). Dashed lines, linear regression (*n=*11 at 25 °C, *P*<0.005; *n*=12 at 33 °C, *P*<0.013) suggesting an increase in the expected distance between the axonal AP-initiation site and the soma. (**c**) Characteristic examples of axonal recordings showing the first (black) and third (red) spikes generated during somatic depolarization (as in Fig. 2c) for a relatively proximal (top) and a relatively distal (bottom) axonal recording site, as indicated. (**d**) Changes in the amplitude (left panel, *n*=30) and the rise time (right panel, *n*=35) of the third compared with the first spike, plotted against the distance from the recording site to the soma. Red lines, best fit single-exponential decay.

We next asked whether somatic depolarization directly controlled by somatic current injection can also affect the AP-initiation region. We therefore compared (*t*_a_−*t*_s_) values for the APs generated following either shorter (0–15 ms) or longer (15–120 ms) intervals of subthreshold depolarization, in the same cell (Fig. 3a). The depolarization rate was controlled by the amplitude of the current injection step applied through a somatic pipette (current-clamp mode): due to the capacitive and active-conductance properties of granule cells, step injection produces gradual depolarization (Fig. 3a). We have found that slowing down the depolarization rate reduced the (*t*_a_−*t*_s_) value progressively with greater distances from the soma (Fig. 3b; *P*<0.005, *n=*11 at 25 °C; *P*<0.013, *n*=12 at 33 °C), similar to the effect observed during repetitive spiking (compare with data in Fig. 2f,g).

### Somatic influence on the Na^+^ channels weakens with distance

The most parsimonious explanation for the use-dependent expansion of the AP-initiation site is that during somatic depolarization the peak density of available Na^+^ channels shifts away from the soma. A basic physiological mechanism that could account for this is partial inactivation of axonal Na^+^ channels because of depolarization propagating into the axon. Two earlier observations support this hypothesis. First, in hippocampal MFs the recovery of Na^+^ channels from depolarization-dependent inactivation could last for seconds^34^ even though their post-spike inactivation recovery is relatively rapid, 10–20 ms^35^. More recently, outside-out patch recordings from axonal patches provided direct characterization of the Na^+^ channel kinetics and its depolarization-dependent inactivation in MFs^29^. Second, depolarization of granule cells readily propagates into the axon showing a distance-dependent decay^25^,^27^. We therefore asked whether we could detect changes in the local axonal spike waveform that would depend on somatic depolarization and the distance from the soma. To achieve this, we compared the waveforms of the first and the third axonal spikes generated by somatic depolarization in the same current trace (Fig. 3c).

As loose-patch axonal recordings represent a mixture of membrane voltage *V*(t) and, predominantly, its first derivative d*V*(*t*)/d*t*^36^,^37^, we considered both amplitudes and rise times of recorded deflections. We found that near the soma the spike amplitude was robustly reduced by the time of the third AP (by 28±6%) and that this reduction decayed with a length constant of 187±108 μm along the axon (mono-exponential fit; *n=*30 soma–axon pairs; Fig. 3d, left). Similarly, by the third AP the spike rise time was increased by 98±23% (that is, by 150–200 μs) near the soma, and this deceleration effect decayed along the axon with a length constant of 43±18 μm (*n=*35; Fig. 3d, right). Whether the recorded axonal spikes represent *V*(*t*) or d*V(t)*/d*t*, or both, these data clearly indicate that somatic excitation reduces the speed with which axonal *V*(*t*) rises during spike generation and that this effect fades away with distance along the axon.

The AP waveform is dictated by the interplay between ion currents through Na^+^ and K^+^ channels. Recent analyses of spiking mechanisms in MFs have indicated that the onset of the K^+^ current is close to the end of the Na^+^ current transient^29^,^38^. Therefore, the changes in the AP rising phase documented here (Fig. 3c,d) should reflect a decrease in Na^+^ channel activation, which is weakening with greater distances from the soma. This phenomenon is likely to reflect a simple (but not demonstrated previously) mechanism by which somatic depolarization partially inactivates axonal Na^+^ channels in a distance-dependent manner. This is because depolarization-dependent inactivation of K^+^ channels should, if anything, affect the decay, rather than rise time, of the AP^28^,^29^,^38^,^39^.

### Somatic depolarization shifts away peak axonal Na^+^ entry

**Figure 4:**
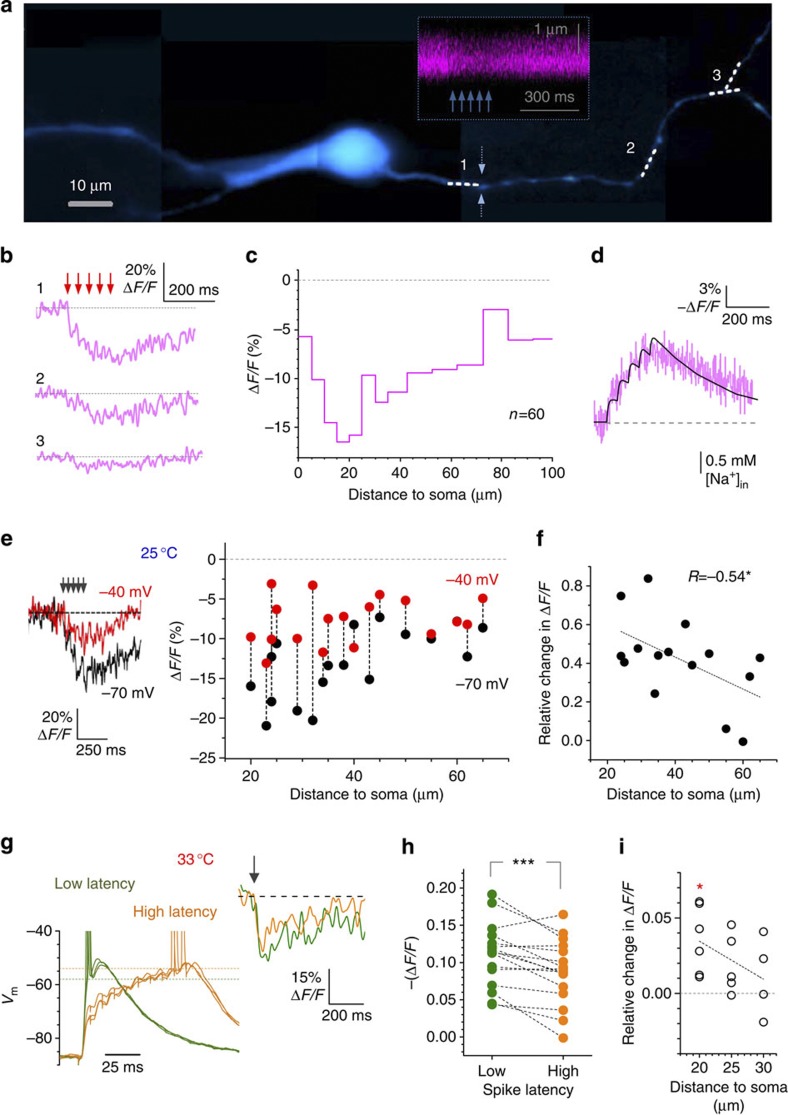
Somatic depolarization flattens the profile of Na^+^ entry along the axon. (**a**) Image: granule cell filled with 1 mM SBFI (for example, collage of averaged 20–30 μm *z*-stacks, *λ*_x_^2*P*^*=*800 nm); dotted segments 1–3, recording sites; inset, example linescan at site 1 (dotted arrows, scan position). (**b**) Characteristic linescan traces in response to five APs at 20 Hz as shown in **a**. (**c**) Axonal distribution of relative Δ*F/F* amplitude (evoked by five APs at 20 Hz). To improve S/N ratio, two largest Δ*F/F* (out of 3–10 traces) within each bin were averaged (*n=*60 recorded sites in total). (**d**) Average SBFI Δ*F/F* signal (magenta, top scale) near the spike-initiation segment (as in **a**,**b**), and the best-fit model representation (black, bottom scale) of Na^+^ entry; see details in Methods and Supplementary Fig. 4. (**e**) Traces: SBFI fluorescence (example) in proximal axon evoked by five spikes at 20 Hz (as in **a**) under baseline conditions (black, −70 mV) and during somatic pre-depolarization (to −40 mV; red). Plot: summary, the SBFI Δ*F/F* amplitude versus distance from the soma (data at >20 μm shown to reduce bias due to somatic Na^+^ diffusion sink); 25 °C. (**f**) Relative changes in Δ*F/F* from experiments in **e**; line, negative linear regression (*n=*15, Pearson’s *R*=−0.54, *P*<0.04). (**g**) Left traces: somatic voltage during electrical stimulation of presynaptic perforant path fibres (as in Fig. 1) with stronger (green) and weaker (orange) stimuli; dotted lines, higher spiking threshold upon slower depolarization. Right traces: SBFI Δ*F/F* recorded simultaneously in the proximal axon (colour-coded accordingly); 33 °C. (**h**) Summary of experiments shown in **g**: colour-coded accordingly; ***P<0.002, *n*=16 cells. (**i**) Summary of experiments shown in **g**: increases in AP-evoked Na^+^ entry SBFI Δ*F/F* amplitude) due to reduced spike latency (faster depolarization) are weaker with greater distances from soma; data average at 20 μm are significantly above zero (*P<0.02, *n*=6, *t*-test); dotted line, best-fit linear slope.

The expression of Na^+^ channel subunits peaks within the MF axon initial segment coincident with the AP-initiation site^32^. To test whether this reflects the distribution of AP-evoked Na^+^ entry along the axon, we imaged axonal Na^+^ entry using the Na^+^-sensitive fluorophore SBFI (*K*_D_~\n25 mM)^40^. As the low-affinity SBFI imposes heavy time filtering and yields a relatively low signal-to-noise ratio for individual spike-evoked Na^+^ signals, we evoked five spikes at 20 Hz using 2-ms depolarizing pulses while monitoring SBFI fluorescence along the axon (Fig. 4a,b). In these experiments, the Na^+^-dependent inverse fluorescence increment indeed peaked 20–25 μm from the soma (Fig. 4c, *n=*60 recorded sites), consistent with the AP-initiation site (Fig. 2d). An explicit kinetic model of Na^+^ entry, together with an independent estimate of the SBFI association constant (*k*_on_~\n2.0 M^−1^ms^−1^), suggested that these data correspond to an ~\n0.4-mM increment of axonal Na^+^ per spike near the AP-initiation site (Fig. 4d; Supplementary Fig. 4). If the depolarization- and distance-dependent inactivation of Na^+^ channels indeed underlies the expansion of the AP-initiation site, then the profile of Na^+^ entry along the axon should change correspondingly. As existing Na^+^ imaging techniques cannot directly measure Na^+^ entry on the submillisecond scale (for spike latency readout), we tested our hypothesis in two alternative types of experiments.

In the first experiment, we used relatively crude somatic voltage manipulation. We monitored axonal Na^+^ entry evoked by a brief train of spikes, with and without the granule cell soma depolarized to −40 mV before the spike onset. We found that depolarization markedly reduced the AP-evoked Na^+^ entry in recorded sites within ~\n70 μm from the soma (Fig. 4e), suggesting overall reduction in Na^+^ channel activation. Importantly, this reduction was consistently weaker with greater distances from the soma (Fig. 4f) reflecting a decaying effect on Na^+^ channels and the flattening of Na^+^ entry along the axon (Fig. 4e, red dots). As these experiments provided only a first-approximation assessment of the physiological scenario in question, in the second experiment we reproduced the protocol depicted in Fig. 1 (at 33 °C) in which the cell-spiking threshold was regulated by the variable synaptic input-induced depolarization. Simultaneously, we imaged Na^+^ entry in the proximal axonal segment, 20–40 μm from the soma (Fig. 4g; Supplementary Fig. 5). The results showed clearly that slowing down the excitatory input (hence elevating the spike threshold) significantly reduced axonal Na^2+^ entry (by 24±7%, *n*=16, *P*<0.002; Fig. 4h), with the effect decaying with greater distances from the soma (Fig. 4i). Taken together, Na^+^ imaging tests thus lent support to our hypothesis suggesting the distance-dependent inactivation of axonal Na^+^ channels upon somatic excitation as a mechanism for the expansion of the spike-initiation site.

### Changes in axonal Ca^2+^ entry point to Na^+^ channel inactivation

To further assess the roles of Na^+^ and K^+^ channels in use-dependent changes of axonal spiking, we set out to monitor axonal Ca^2+^ entry. This approach has a simple rationale. As Ca^2+^ channels themselves undergo little or no inactivation in conditions of our experiments, axonal Ca^2+^ entry depends almost entirely on the kinetics of Na^+^ and K^+^ channels that shape the AP: partial inactivation of Na^+^ channels should either have little effect or *decrease* Ca^2+^ entry, whereas partial inactivation of K^+^ channels should *increase* Ca^2+^ entry due to the spike-broadening^28^,^39^. Thus, if K^+^ channel inactivation dominated during somatic depolarization, we should observe an increase in Ca^2+^ entry as depolarization progresses, with such an increase being the greatest near the soma (where depolarization is at its strongest).

**Figure 5:**
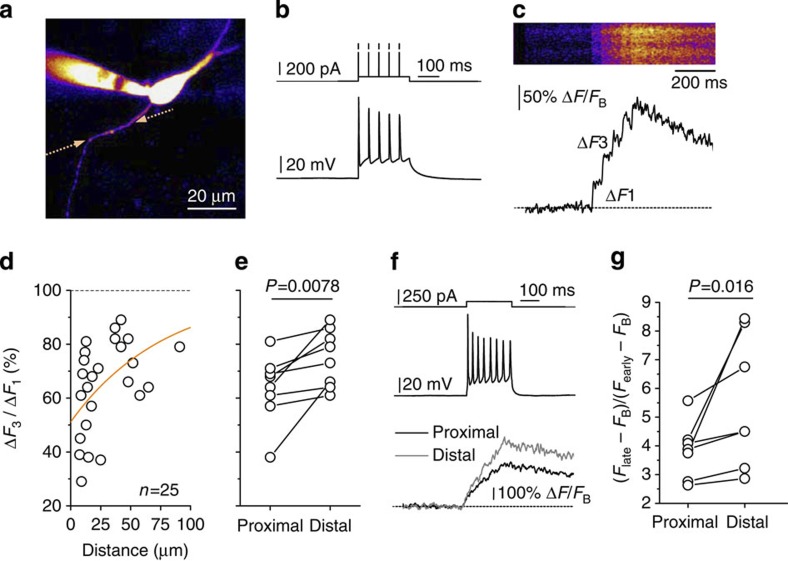
Somatic excitation inhibits axonal Ca^2+^ entry preferentially in proximal segments. (**a**) A typical fluorescence image of a dentate gyrus granule cell (Alexa Fluor 594, *λ*_x_^2*P*^*=*800 nm) with a proximal axonal fragment imaged in linescan mode (dotted arrows, linescan position). (**b**) A 250-ms depolarizing current superimposed with current pulses (0.5–1 ms, 1–2 nA, 20 Hz; top trace) induces a burst of regular spikes (bottom trace). (**c**) Characteristic Ca^2+^ transients recorded in an experiment shown in **a**,**b**; eight trial average; top, original linescan; bottom, quantified trace. Δ*F*_1_ and Δ*F*_3_ denote Ca^2+^-dependent fluorescence increments evoked by the first and the third spikes, respectively. (**d**,**e**) The ratio Δ*F*_3_*/*Δ*F*_1_ is lowest near the soma and increases progressively with the distance along the axon (**d**; *n=*25, orange line: exponential fit). In eight cases (**e**), recordings were made from proximal and distal sites on the same axon allowing direct paired-sample comparison (Wilcoxon signed-rank test; in this sample, the average distance to the proximal site was 38.9±5.9 μm and between proximal and distant sites was 14.9±1.6 μm). Throughout the sample, Δ*F*_1_/*F*_B_ (*F*_B_ stands for baseline fluorescence) showed no correlation with the distance from the soma (Spearman rank test *P=*0.556, *n=*25). (**f**) An example of cell spiking evoked by a depolarizing current (upper trace) and the average Ca^2+^ fluorescence responses in proximal and distal axonal sites (lower trace, *n=*7); note that the spike-evoked fluorescence increments are smoothed out in the average trace because of the jitter in spike timing. (**g**) A summary of experiments shown in **f**; the average distance from the soma to the proximal site was 32.4±12.8 μm and the distance between proximal and distant sites was 10.7±1.4 μm (mean±s.e.m.). *F*_B_, *F*_early_ and *F*_late_ denote, respectively, baseline Ca^2+^ fluorescence and the fluorescence signal integrated over 0–40 and 40–200 ms post onset. In these recordings, *F*_B_ showed no distance dependence (*P=*0.69, *n=*7, Wilcoxon signed-rank test).

We therefore loaded granule cells with a Ca^2+^ indicator (200 μM Fluo-4; Fig. 5a) and carried out two complementing experiments. In the first experiment, we evoked brief trains of spikes by injecting a subthreshold depolarizing current superimposed with 1-ms pulses (at 20 Hz, current clamp; Fig. 5b,c). This protocol provided time-locked APs across the experiments, also enabling us to compare spike-evoked Ca^2+^ entry at different points along the axon. Previously, we showed that in these conditions the increment of Fluo-4 fluorescence Δ*F* evoked by a spike gives a robust indicator of Ca^2+^ entry^41^. In these experiments, the ratio between Δ*F* increments during the third and the first spikes (Δ*F*_3_/Δ*F*_1_) was lowest near the soma while increasing substantially with more distal recording sites, be the data analysed as one sample (Fig. 5d) or compared in one-axon pairs (Fig. 5e). A qualitatively identical conclusion was obtained in the complementing experiment in which the soma was progressively depolarized to generate a brief train of spikes (with some expected spike onset jitter), with Ca^2+^ monitored at two axonal sites (Fig. 5f,g). We also confirmed that our Ca^2+^ data could not be explained by the distance-dependent Ca^2+^ indicator saturation because Ca^2+^ entry (Δ*F* ) did not correlate with the distance to the soma over the measured intervals (*P=*0.556, *n=*25, Spearman rank test), unlike in some other central neurons^42^. Our observations were thus diametrically opposite to those expected if inactivation of K^+^ channels was to play a dominant role here (see above). By exclusion, we were left with the only plausible explanation for our findings: somatic excitation partly inactivated axonal Na^+^ channels, in a distance-dependent manner.

### Biophysics predicts expansion of the spike-initiation site

**Figure 6:**
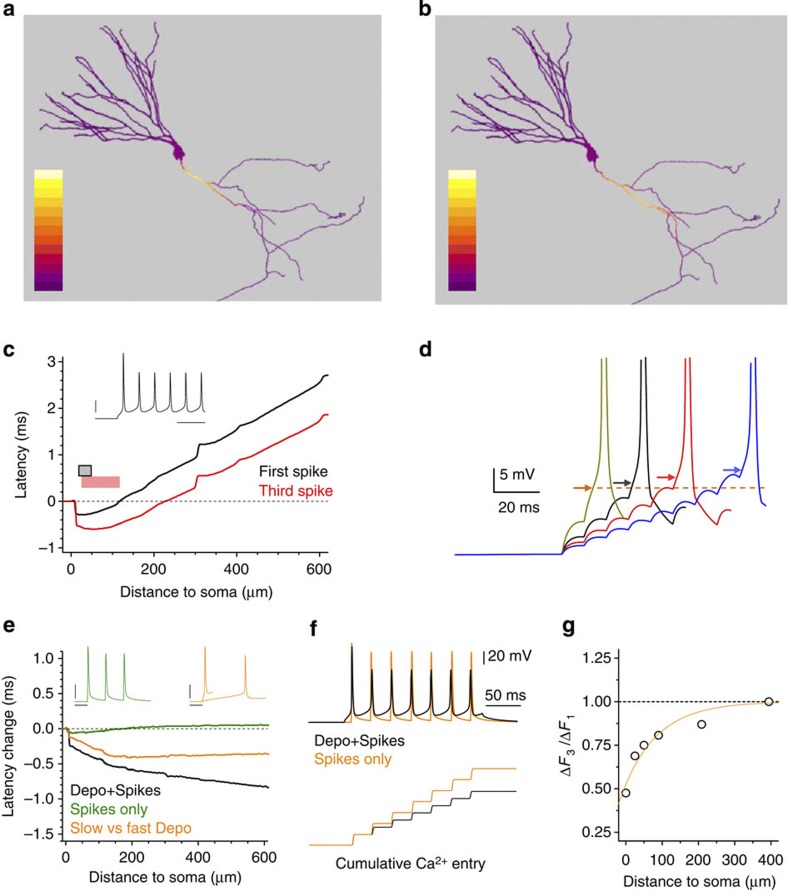
A NEURON granule cell model points to excitation-dependent inactivation of Na^+^ channels. (**a**) A modelled granule cell adapted from Schmidt-Hieber *et al.*^54^ and further complemented as in Scott *et al.*^25^ The colour-coded diagram (d*V/*d*t* values, colour scale bar 0–400 Vs^−1^) shows a snapshot at the time point following somatic depolarization (130 pA injection current) when the maximum depolarization rate ((d*V/*d*t*)_max_, the apparent spike-initiation onset) is reached anywhere in the axon. The area associated with the AP initiation can be seen in the initial axonal segment. (**b**) The snapshot as in **a** but during the third consecutive spike: the area associated with AP initiation can be seen clearly expanding away from the soma (colour scale bar 0–250 Vs^−1^). (**c**) The (*t*_a_−*t*_s_) latency plot obtained as in Fig. 2d for the model shown in **a**,**b**. Colour bars show typical sites of initiation for the first (grey) and third (red) spikes (axonal areas corresponding to a range within ~\n20% of the most negative latency values), as indicated. Scale bar for insets: 20 mV, 50 ms. (**d**) Somatic injection of synaptic current trains of varying intensity in the model demonstrates adaptive properties of the spiking threshold consistent with experiments shown in Fig. 1; arrows, spiking threshold determined as min(d^2^*V*/d*t*^2^), *V*_rest_=−79 mV, dotted line, first spike threshold. (**e**) The somatic excitation-induced change in (*t*_a_*−t*_s_) latency (an indicator for alterations of the AP-initiation site) depends on depolarization and on the distance to the soma, rather than on spiking *per se*. In the three simulation examples, APs were induced by suprathreshold somatic depolarization (Depo+Spikes, black), and by 0.5 ms pulses only (Spikes only, green), and by constant subthreshold depolarization of varying intensity (Slow versus fast Depo, orange), as indicated. Scale bars for insets, 30 mV, 20 ms. (**f**) An example of the simulated AP-induced Ca^2+^ influx during somatic depolarization: such computations were made at different points throughout the axon. (**g**) The ratio of Ca^2+^ increments evoked by the third and first consecutive APs is lowest near the soma (~\n50%) but reduces with larger distances to the soma. This is consistent with experimental observations (Fig. 5) implicating partial inactivation of Na^+^ channels depending on somatic depolarization and distance from the soma. Line, single-exponent fitting.

To test whether partial Na^+^ channel inactivation in proximal axonal segments due to somatic depolarization could provide a self-sufficient mechanistic explanation for our observations, we used a detailed NEURON model of a morphologically reconstructed dentate granule cell^31^. In this model, the dendritic electric properties were exhaustively tested^31^, and the basic axon parameters were evaluated in our earlier dual-patch study^25^ (Methods). As we aimed to understand the mechanism in principle, we focused on the distribution of Na^+^ and K^+^ channel conductance only, in correspondence with our imaging data and the immunofluorescence results reported earlier^32^ (Methods, Supplementary Fig. 6a,b). In granule cells, axonal and somatic Na^+^ channels differ in their inactivation properties^35^, and therefore both channel types^31^,^35^ were incorporated correspondingly in our model (Supplementary Fig. 6a,b). APs elicited in this model could be seen to be initiated in the proximal axon (Fig. 6a), in good agreement with our experimental observations.

Simulations predicted that the depolarization-dependent inactivation of Na^+^ channels could indeed alter AP generation in the axon. As cell spiking progresses, APs begin to initiate over a wide axonal region, rather than within a narrow proximal site (note relatively shallow and expanded false-colour gradients in proximal axonal segments in Fig. 6b compared with Fig. 6a; for the sake of clarity, no stochastic component mimicking experimental observations of spike initiation was included). In the same model, injecting a train of modelled synaptic currents of varying intensity in the soma produced adaptive changes in the cell-spiking threshold (lower threshold with steeper excitation; Fig. 6d), which were fully consistent with our experimental observations (Fig. 1). Equipping the model with the recently updated MF Na^+^ channel kinetics^29^ produced a similar outcome indicating a robust nature of this phenomenon (Supplementary Fig. 6c). Correspondingly, subthreshold depolarization prominently inactivated Na^+^ channels resulting in a substantial latency shift (orange line, Fig. 6e).

This was consistent with the spike-initiation site expanding along the axon, away from the soma. To confirm that the effect was not a spurious consequence of post-spike relaxation phenomena in the model, we forced spike trains without depolarization (by generating 0.5-ms pulses at resting voltage). Spiking without depolarization resulted in little Na^+^ channel inactivation during the train, no latency changes between the first and third consecutive spikes, and hence no changes in the spike-initiation site (green line in Fig. 6e). Next, we introduced voltage-gated Ca^2+^ channels in the model (generic VGCCs adapted from the study by Royeck *et al.*^43^). This prompted the model to reproduce not only the recorded progressively decreasing Ca^2+^ entry near the soma during depolarization-induced spiking (Fig. 6f) but also the weakening of this effect with greater distances from the soma (Fig. 6g), in accord with our experimental observations (Fig. 5). Finally, an artificial increase in depolarization-dependent K^+^ channel inactivation in the model predicted changes in use-dependent Ca^2+^ entry (Supplementary Fig. 7), which were opposite to what we recorded (Fig. 5), thus again confirming the predominant role of Na^+^ channel inactivation. Taken together, our simulations with the realistic granule cell model, complemented by Monte Carlo tests dealing with a stochastic component of spike initiation (Fig. 2h, Supplementary Fig. 2), could thus reproduce and mechanistically explain our experimental observations made throughout the study.

## Discussion

The main findings of this study are as follows. First, we showed that hippocampal granule cells, similar to many other neurons in the CNS, can adapt their spiking threshold to the rate of the excitatory glutamatergic input (enacted by perforant path synapses). The extent of this adaptation is significant: slowing down the synaptic input could have the same effect on cell firing as 20–30% of the total presynaptic input. Consequently, stronger synchronization of perforant path inputs is likely to have an advantage in terms of firing the cell compared with less synchronized inputs that produce the same cumulative excitation. The adjustment of cell excitability to physiological activity thus occurs in real time, on the scale of dozens of milliseconds. To understand the underlying mechanism, we asked how the somatic depolarization affects spike initiation in the initial axonal segment. It was recently reported that even small plastic changes in the availability or distribution of Na^+^ channels in this segment may have a profound effect on cell excitation^16^,^21^.

We found that, under resting conditions, the MF spike-initiation site was 20–25 μm from the soma, consistent with earlier observations^31^,^32^, whereas APs propagated reliably along the MFs, up to the maximal firing frequency. The latter finding suggests that use-dependent short-term plasticity of MF transmission^24^,^44^ is unlikely to involve AP propagation failures, at least under baseline conditions. More importantly, during somatic excitation, the area of AP initiation *broadens* dramatically along the axon. This broadening is paralleled by the reduction in the rate of voltage rise, and consequently in the apparent propagation speed, of local axonal spikes: the effect was greatest in most proximal axonal segments and faded away at larger distances from the soma. In theory, having a broad AP-initiation site could be interpreted as APs starting to generate ‘quasi-simultaneously’ over a relatively large axonal region. A more realistic scenario, however, is that individual APs are initiated stochastically at various sites along the initiated region. Indeed, stochastic AP generation is expected to increase the variability for soma–axon spike latencies, which is fully consistent with our data indicating that the trial-to-trial variability of (*t*_a_−*t*_s_) increases during the spike train (also reflected in the increased variability across the sample and suggestive of local spike slowdown). This observation proposes that the probability of AP initiation becomes spread more evenly along the proximal axon in the course of somatic depolarization. In line with this suggestions, Monte Carlo simulations of latency measures predicted use-dependent broadening of the proximal axonal segment, apparently with a contribution of spike deceleration in proximal segments^32^.

The most parsimonious cellular mechanism underlying these observations was partial inactivation of axonal Na^+^ channels by somatic depolarization, which propagates electrotonically into the axon. Although this hypothesis appears to be a straightforward theoretical consequence of voltage-dependent channel kinetics and electrotonic propagation of somatic voltage into the axon, it has not been explored previously, either theoretically or empirically. To test this hypothesis further, we imaged axonal Na^+^ and found that somatic depolarization (induced by direct current injection or via dendritic synaptic inputs) reduced AP-evoked axonal Na^+^ entry, with the greatest effect in more proximal segments. This was fully consistent with the distance-dependent Na^+^ channel inactivation.

Another piece of experimental evidence implicating depolarization-dependent inactivation of Na^+^ channels was obtained using axonal Ca^2+^ imaging. We have found that somatic excitation progressively reduces Ca^2+^ entry and that this effect weakens with the distance from the soma. This was opposite to what was expected from K^+^ channel inactivation that increases Ca^2+^ entry upon depolarization^28^,^39^,^45^,^46^. We concluded, therefore, that depolarization- and distance-dependent partial inactivation of Na^+^ channels dominates in modulating the AP-dependent Ca^2+^ entry in proximal MF segments. It remains an intriguing and open question whether our Ca^2+^ imaging results could also implicate use-dependent plasticity of local Ca^2+^ entry in the spiking mechanism control more directly, via the molecular cascades described recently^42^.

Interestingly, our previous observations indicated that somatic depolarization of granule cells could increase spike-evoked Ca^2+^ entry in proximal axonal segments^26^. However, these earlier experiments were carried out by distantly evoking antidromic spikes while holding the soma in voltage clamp. This must have curtailed depolarization-dependent Na^+^ channel inactivation, thus in effect reproducing the ‘Spikes only’ scenario in our present study (Fig. 6e,f), probably through facilitation of depolarization-dependent K^+^ channel inactivation. Finally, we asked whether our observations were consistent with the biophysics of spike generation and propagation in granule cell axons. We therefore explored a previously tested NEURON model of a fully reconstructed granule cell including the axon. Throughout the study, we were able to readily reproduce our experimental observations in a detailed compartmental model of a three-dimensional (3D) reconstructed granule cell, demonstrating a mechanistic link between distance-dependent Na^+^ channel inactivation, expanded spike-initiation site, changes in axonal Ca^2+^ entry and use-dependent adaptation of the cell-spiking threshold.

Synchronization of spiking activity among principal neurons has been thought as an essential part of neuronal information handling^47^ including conscious processing^48^. Our results in hippocampal granule cells lend further support to the view that the simple sum of excitatory inputs can produce different outcomes depending on the time course of summation, with highly synchronized inputs gaining an added effective weight^7^,^8^,^9^. Previously, the differences in geometry and position of the axonal AP-initiation site were found to underlie adaptation to particular spiking patterns among individual neurons in the *nucleus laminaris* of birds^19^. The present findings advance this concept further, suggesting that the spike-initiation region could be considered as a plastic rather than a fixed entity, not only in terms of long-term developmental or adaptive changes but also during the ongoing network activity. In line with this hypothesis, a very recent study has found that subtle use-dependent changes in the local Na^+^ channel density and therefore spike-initiation site in cortical neurons could have a large influence on neuronal excitability^16^. It would be important to understand how universal this mechanism is among excitatory circuits of the brain.

## Methods

### Electrophysiology

All animal experimentation routines followed the UK Animals Scientific Procedures Act (1986) amended and adapted to comply with the European Directive 2010/63/EU on the Protection of Animals used for Scientific Purposes. Acute 350-μm hippocampal slices from 3- to 4-week-old male Sprague–Dawley rats were transferred to a submersion-type recording chamber (Scientific Systems Design, NJ, USA) and superfused with (mM) 124 NaCl, 2.5 KCl, 2 CaCl_2_, 1.3 MgSO_4_, 26 NaHCO_3_ and 10 glucose and were bubbled with 95% O_2_/5% CO_2_. The slice orientation that helps to avoid cutting MFs was detailed previously^41^. The internal solution included (mM) the following: 135 K methanesulfonate, 2 MgCl_2_, 10 HEPES, 10 di(Tris)-Phosphocreatine, 4 NaATP, 0.4 NaGTP and fluorophores as indicated. Dentate granule cells were discarded if their resting potential increased above −70 mV in current clamp mode. Receptor antagonists were purchased from Tocris Cookson, and fluorescent probes were purchased from Invitrogen. We used the procedures detailed earlier^25^ to track and record from granule cell axons. In brief, we traced axons in the Alexa emission channel (up to 150–200 μm deep in the slice) and approached a selected fragment with a patch pipette, in voltage-clamp configuration, applying negative pressure to achieve loose patch (100–200 MOhm seal resistance). Experiments were carried out either at ~\n25 or ~\n33 °C, as indicated.

### Two-photon excitation fluorescence imaging

We used a Radiance 2000 or 2100 (Zeiss-Bio-Rad) imaging system optically linked to a MaiTai (SpectraPhysics-Newport) femtosecond-pulsed infrared laser (in London), a FV10-MP (Olympus) system linked to a Vision-S (Coherent) laser (in Bonn) or a confocal scanning unit (LCS Leica SP5 II, Leica Microsystems, Mannheim, Germany) attached to an upright microscope (DM6000CFS, Leica Microsystems; 25 × objective, HCX IRAPO L 25 × , numerical aperture 0.95, Leica Microsystems) linked to a MaiTai laser (in Alicante). The imaging systems were integrated with patch-clamp electrophysiology. Single orthodromic escape action currents were evoked by a 2-ms command voltage pulse. Recording sweeps (normally 500-ms long) were collected at 5 or 20 kHz (dual-patch recordings) with 30-s or 1-min intervals. Granule cells held in whole-cell mode were loaded with two fluorophores: a morphological tracer Alexa Fluor 594 (20–40 μM) and either the low-affinity Na^+^ indicator SBFI (1 mM) or the high-affinity Ca^2+^ indicator Fluo-4 (200 μM). Fluorophores were excited in two-photon mode at *λ*_x_^2*P=*^800 nm. The technique to trace MFs into the CA3 region 500–1,000 μm from the soma has been detailed earlier^41^. The axon was imaged at the maximal optical resolution (~\n0.2 μm, 70 nm per pixel). Experiments involving axon tracing and imaging were carried out at 25 °C or 33 °C, as indicated. The Ca^2+^-dependent Fluo-4 fluorescence signal (Fig. 5) was quantified as Δ*F*/*F*_B_=(*F*−*F*_pre_)/(*F*_pre_−*F*_0_), where *F*_0_ denotes the background fluorescence and *F*_B_=*F*_pre_−*F*_0_ the pre-stimulus baseline axonal fluorescence. For Ca^2+^ transients evoked by precisely timed spikes generated in response to brief depolarizing pulses (Fig. 5c–e), the ratio of Ca^2+^-dependent fluorescence increments during the spike train are given as Δ*F*_3_/Δ*F*_1_ for the third and first spikes. When Ca^2+^ transients were elicited by depolarization-induced and therefore variable spike trains (Fig. 5f,g), the ratio of early fluorescence increments *F*_early_ (0–40 ms after stimulus onset) and delayed increments *F*_late_ (40–200 ms after stimulus onset) was calculated: (*F*_late_−*F*_B_)/(*F*_early_−*F*_B_).

Action potential-evoked Na^+^ entry in response to synaptic input (Fig. 4g–i) was estimated from axonal SBFI fluorescence 10–30 μm away from the soma. SBFI fluorescence was first normalized to that of the sodium-insensitive morphological marker Alexa Fluor 594 and then normalized to baseline. Individual fluorescence traces with low or high AP latency, three to five trials for each, were then aligned at the AP peak and averaged. Image analysis was performed on stacks of stored linescan images using *ImageJ (NIH)* macros or in Matlab (Mathworks). False-colour tables and averaged images were used for illustration purposes but the quantitative analyses always dealt with the original (grey level) pixel brightness values. In most experiments, we reconstructed the axon trajectory using a collage of high-resolution Kalman-filtered z-stacks 15–20 μm deep using 10-scan average frames.

### Quantifying axonal Na^+^ entry

To translate SBFI (sodium-binding benzofuran isophthalate) fluorescence into the internal Na^+^ concentration ([Na^+^]_in_) time course, we considered the explicit kinetics of Na^+^ entry, binding and extrusion, by adapting the multicomponent single-compartment kinetic model, which was extensively tested earlier^41^ (see below). As such models do not assume steady state and thus require the knowledge of the indicator binding on-rate, 
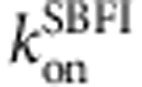
 (in addition to the known *K*_D_), we attempted to estimate its value first. To achieve this, we exploited the fact that the typical rise time of SBFI fluorescence following a single spike is 20–30 ms, whereas active Na^+^ extrusion mechanisms act on a much slower timescale. Indeed, classical measurements in giant axons^49^ indicate the maximal achievable axonal Na^+^ efflux rate in the range of 20–40 pmol cm^−2 ^s^−1^, which translates into 1.6–3.2 mM s^−1^ for a ~\n0.5-μm-wide axon (comparable to MFs), in agreement with measurements in granule cell axons^50^. In other words, Na^+^ extrusion post spike should have a negligible effect on [Na^+^]_in_ time course during spike-evoked Na^+^ influx (1 ms scale) or Na^+^ binding to SBFI (20- to 30-ms scale). Furthermore, a multicompartmental model of a granule cell^41^ predicted that Na^+^ equilibration post spike because of diffusion along the axon within 20- to 30-ms post entry should have little influence (<5%) on the longitudinal [Na^+^]_in_ profile (not shown). These considerations allowed us to constrain the [Na^+^]_in_ kinetic, for at least 20–30 ms post entry, to a single free parameter, 
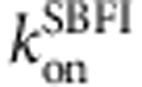
. We therefore imaged SBFI fluorescence near the AP-initiation site in response to a somatic voltage step from −75 to +10 mV (thus evoking a very brief pulse of Na^+^ entry through rapidly inactivating Na^+^ channels). The rising phase kinetics of the SBFI-dependent fluorescence signal was best fitted with 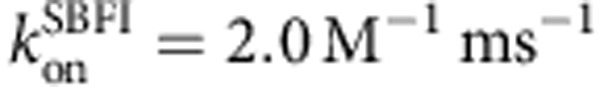 (Supplementary Fig. 3a).

Equipped with this value, we were able to evaluate the remaining kinetic parameters, the Na^+^ extrusion rate *P*_Na_ and the amount of Na^+^ entry expressed as the spike-evoked [Na^+^]_in_ increment Δ*N*a. To achieve this, we recorded SBFI fluorescence near the AP-initiation site under two complementary protocols, with five APs at 20 Hz and 100 APs at 50 Hz (by applying 2-ms depolarizing pulses), as detailed earlier^41^,^51^. On the timescale of these experiments, residual Na^+^ escape because of the [Na^+^]_in_ gradient along the axon was well approximated by a first-order removal rate of ~\n0.8 s^−1^ (Supplementary Fig. 3b,c). The remaining two adjustable parameters, ΔNa and *P*_Na_, were therefore reasonably constrained between the two protocols. Furthermore, the influences of ΔNa and *P*_Na_ on the [Na^+^]_in_ kinetics were in effect mutually independent (that is, orthogonal) over the tested physiological range: ΔNa scaled with the fluorescence (inverse) increment signal Δ*F/F*, whereas *P*_Na_ defined predominantly the signal (inverse) decay. The fitting procedure was therefore relatively straightforward, giving the best fit at *P*_Na_~\n0.0022, ms^-1^ and ΔNa~\n0.4 mM, which was in correspondence with the axonal Na^+^ entry estimated in cortical pyramidal cells^12^. These estimates enabled us to calculate the [Na^+^]_in_ time course near the AP-initiation site (Supplementary Fig. 3b,c).

### Multicomponent model of local Na^+^ dynamics

To evaluate the intra-axonal Na^+^ kinetics based on SBFI fluorescence data, we used an explicit finite-difference kinetic scheme^41^,^52^ incorporating Na^+^ entry rate *j*_Na_, the binding–unbinding reactions with the indicator *SBFI,* and the Na^+^ removal rate *P*_Na_:


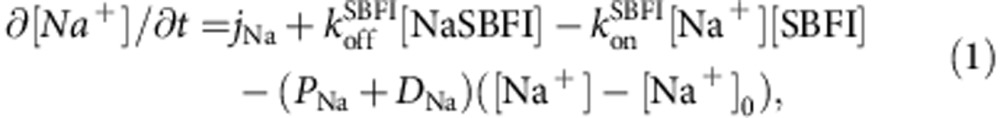


with the mass conservation rules:


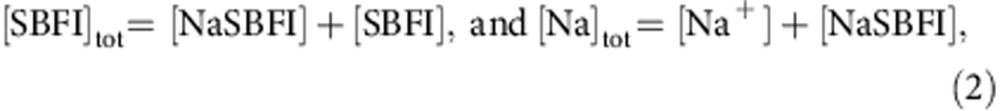


where brackets denote concentrations, NaSBFI stands for the Na-bound indicator, *P*_Na_ in equation (1) is the Na^+^ extrusion rate, *D*_Na_ in equation (1) is its longitudinal diffusion escape rate and index ‘tot’ denotes total amount.

The AP-evoked Na^2+^ influx rate *j*_Na_ was approximated by the Gaussian^52^,^53^


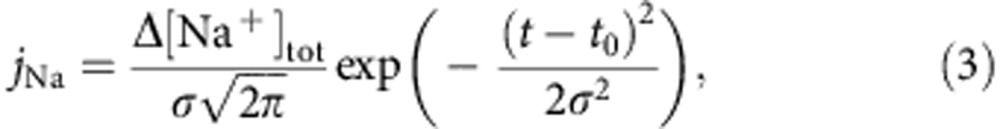


roughly representing the AP waveform, with the MF spike half-width of *σ*≈0.5 ms (ref. 39), onset at *t*_0_ and the time integral of Δ[Na^+^]_tot_ reflecting total Na^+^ entry. Twofold variations in *σ* values in equation (3) had negligible influence on the kinetics of Na^+^–SBFI binding.

Once the 
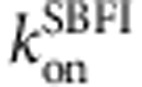
 and the range of *D*_Na_ have been established (the effect of *D*_Na_ was negligible, data not shown), the model operated with only two adjustable (free) parameters: Δ[Na^+^]_tot_ and *P*_Na_. However, varying either Δ[Ca^2+^]_tot_ or *P*_Na_ had virtually independent effects on, respectively, the calculated amplitude (Δ*F/F*) and decay of fluorescent responses. Each of the two parameters could be, therefore, constrained by a straightforward fitting procedure that would match the calculated and the experimental fluorescence, as detailed earlier^41^.

### Compartmental NEURON model of a dentate granule cell

We used a NEURON model of a fully reconstructed dentate granule cell^54^ (cell no 7 in the cited reference), which could be directly imported from the database at SenseLab, ModelDB=95960 at http://senselab.med.yale.edu/modeldb. Passive axon parameters were adopted from measurements made earlier^25^. Axonal axial resistance was set to 80 Ohm cm. Sodium and potassium channel kinetics was implemented as described^31^ and distributed as a function of distance from the soma (Supplementary Fig. 6). The channel conductance distribution was adjusted to reproduce experimental observations (without introducing additional membrane conductances). The reverse potentials for Na^+^, K^+^ and leak currents were set to 58, −95 and −80 mV, respectively. Axon collateral diameters were set at 0.25 μm, their axial resistance was 300 Ohm cm and their maximum conductances for Na^+^ and K^+^ were, respectively, 0.012 and 0.003 S cm^−2^. Action potentials were elicited by somatic current injections as illustrated. In accord with experimental measurements, AP initiation in a particular section of the cell was considered to occur at the time when the rate of depolarization (d*V*/d*t*) reached its maximum value (d*V*/d*t*)_max_. The site of AP initiation was defined as the first (earliest) section initiating an AP in response to current injection. A latency plot equivalent to experimental protocols was calculated using the spatial distribution of AP-initiation onsets (Fig. 6). To monitor Ca^2+^ influx along the axon, a generic Ca^2+^ channel model was adopted from the study by Royeck *et al.*^43^ (ModelDB, accession: 115356) and distributed homogeneously at a relatively low density (100 nS cm^−2^). As our simulations were focused on the total Ca^2+^ entry (increments) rather than the kinetics of free Ca^2+^, intracellular Ca^2+^ buffering and extrusion were ignored in the model.

## Author contributions

R.S.S., C.H., R.P., S.A. and T.P.J. performed experimental studies and analyses, C.H. and D.A.R. carried out modelling studies; D.A.R. narrated the study, which was subsequently contributed by all the authors.

## Additional information

**How to cite this article:** Scott, R. S. *et al.* Neuronal adaptation involves rapid expansion of the action potential initiation site. *Nat. Commun.* 5:3817 doi: 10.1038/ncomms4817 (2014).

## Supplementary Material

Supplementary Information Supplementary Figures 1-7 and Supplementary References
