# Supplementary material for: Neuronal adaptation involves rapid expansion of the action potential initiation site
Source: Nat Commun. 2014 May 23;5:3817. doi: 10.1038/ncomms4817 (PMC4050282; doi:10.1038/ncomms4817)
Supplement: Supplementary Information — Supplementary Figures 1-7 and Supplementary References [file ncomms4817-s1.pdf]

Supplementary Information

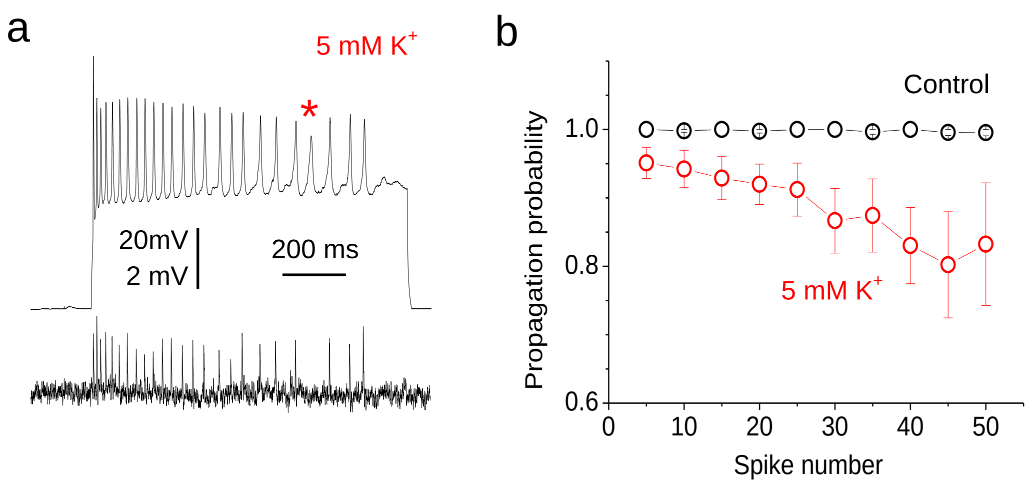

Supplementary Figure 1 A physiologically relevant increase in extracellular potassium reduces the spike propagating fidelity at mossy fibers.

(a) Characteristic traces showing action potentials recorded at the granule cell soma upon a depolarizing pulse (whole-cell current clamp) and spike deflections recorded at the axonal bouton, ~400 µm from the soma (lower trace; loose-patch current clamp).

(b) Statistical summary of experiments illustrating propagation fidelity of APs, depending on the total spike number, generated by an 800 ms depolarizing pulse; black circles, recordings in baseline conditions (data from n = 7 main axons and n = 6 collateral branches were indistinguishable and therefore combined), all mean values are >0.995; red circles, recordings with 5mM KCl added to the bath solution (n = 7); dots, mean ± s.e.m. See Fig. 2b for an example from the same cell recorded in baseline (control) conditions.

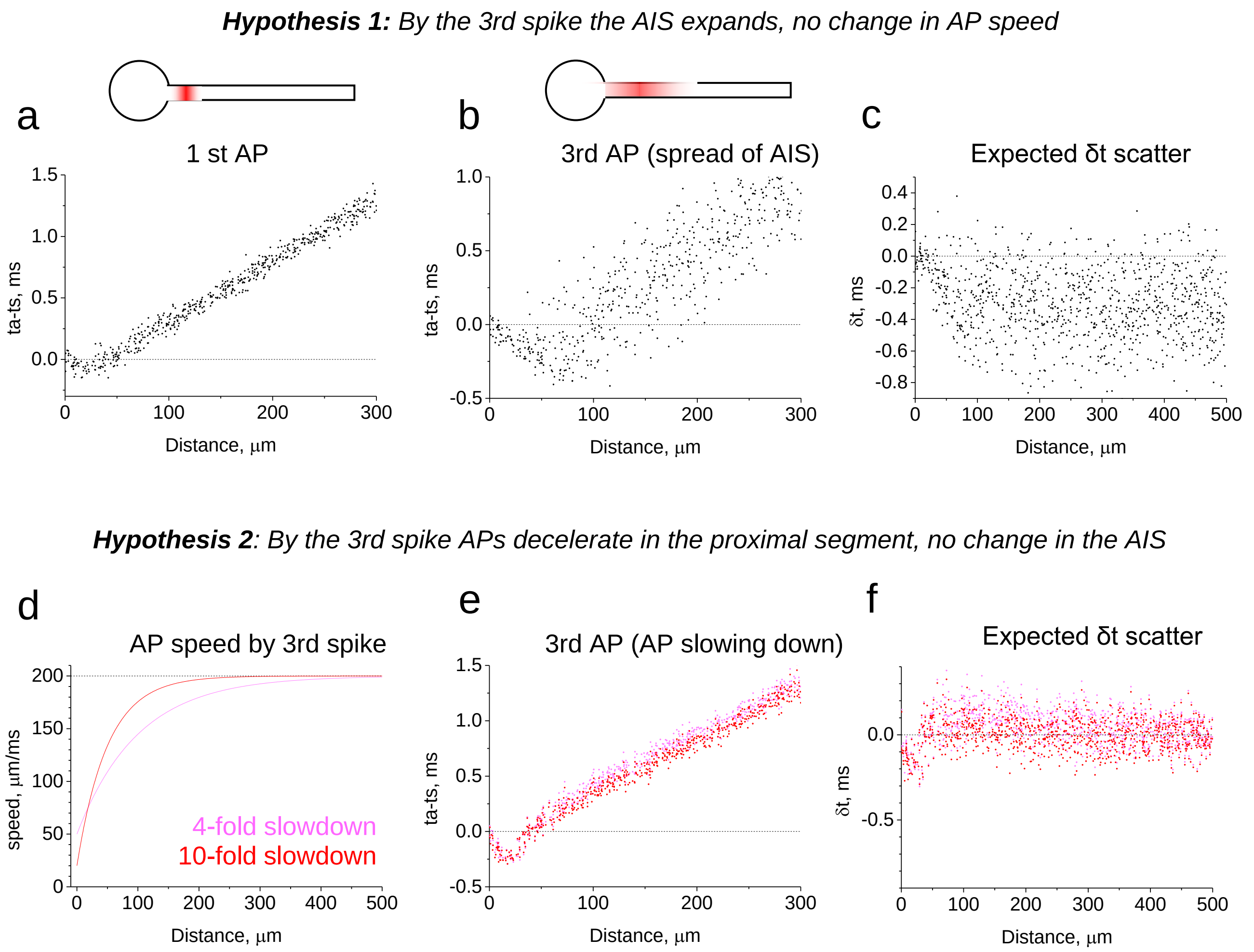

Supplementary Figure 2 Theoretical predictions based on either the initiation site expansion or the spike deceleration in the proximal axon, both resulting from somatic depolarization.

(a) Monte-Carlo simulated (t_a - t_s) atency values (as in a), with action potentials (APs) initiated stochastically at 20 ± 2 µm (Gaussian: mean ± σ) from the soma, representing resting condition (inset diagram, red shade representing the AP initiation site, AIS, in terms of spike initiation probability; not to scale). 1000 trials, axonal recording points are scattered evenly at 0-500 µm (or 0-750 µm in Fig. 2h example); AP propagation speed, 200 µm/ms; latency measurement error / noise: 50 µs (Gaussian).

(b) Monte-Carlo simulated (t_a - t_s) latency values as in (a), with action potentials (APs) initiated stochastically at 50 ± 20 μm (Gaussian: mean ± σ) from the soma, representing post-depolarization condition (or 3rd spike; inset diagram, red shade representing AP initiation probability; not to scale). Other parameters are as in (a).

(c) Simulated data scatter of the (t_a - t_s) latency changes (δt) between first (resting condition) and third (post-depolarization) spike, as in Fig. 2f-g.

(d) Simulated four-fold (magenta) and ten-fold (red) deceleration of APs (from the original 200 μm/ms) in the axon proximal segment upon somatic depolarization, with the length constants of speed recovery of 100 μm and 50 μm, respectively.

(e) Monte-Carlo simulated (t_a - t_s) latency values as in (a), with action potentials (APs) initiated stochastically at 20 ± 2 μm (Gaussian: mean ± σ) from the soma, as in resting conditions, but with the AP speed changes as shown in (d), color-coded accordingly. Other parameters are as in (a).

(f) Simulated data scatter of the latency changes (δt) between first (resting condition, as in a) and third (post-depolarization) spike, for conditions described in (d) and (e), color-coded accordingly.

Note: Simulated data represent characteristic examples; the outcome remains qualitatively robust over several-fold changes in the Gaussian distribution parameters of AP initiation, in AP speed values, or in the degree of AP deceleration.

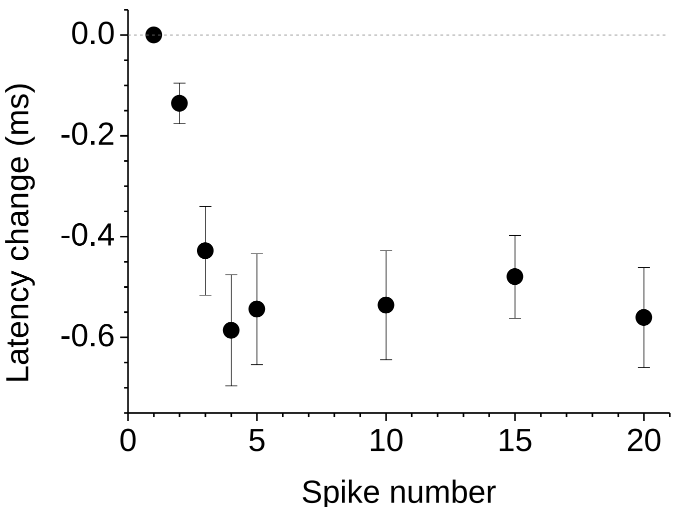

Supplementary Figure 3 Cell excitation broadens the axonal spike initiation site.

The depolarization-dependent expansion of the AP initiation site is reflected in spike latency changes (ordinate), with the effect reaching its maximum approximately by the third spike; average data for $n = 8$ axons (mean ± s.e.m.). The distance from the axonal recording site to the soma was 45-490 μm.

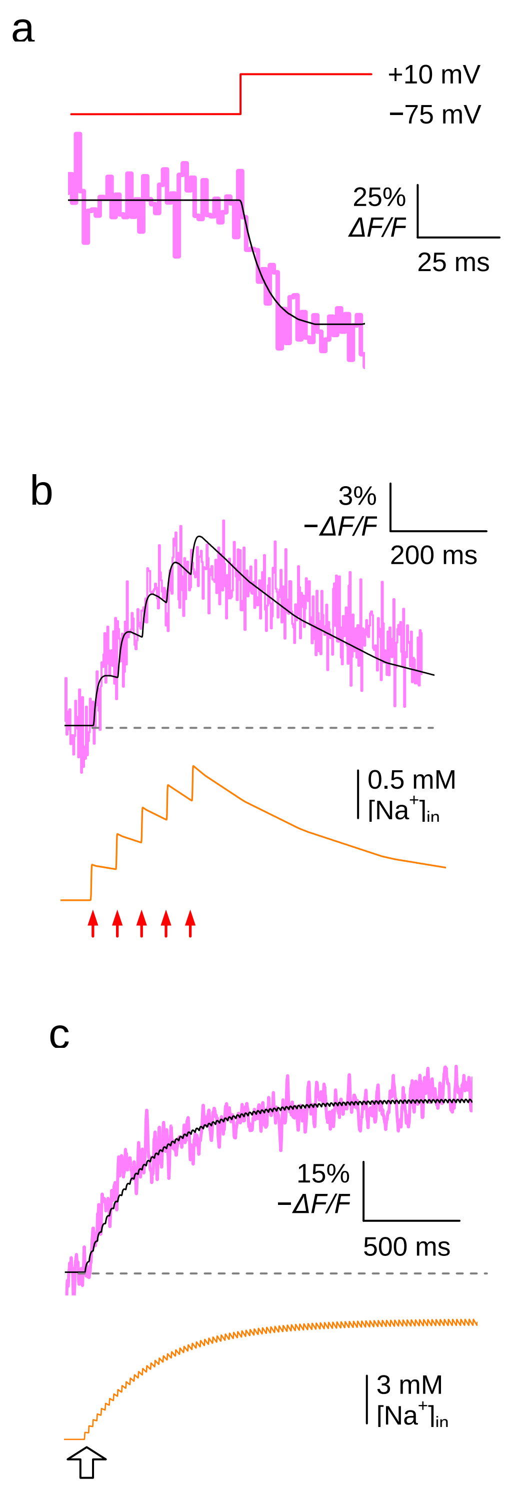

Supplementary Figure 4 Quantitative analyses of Na^+ imaging in granule cell axons

(a) The average SBFI fluorescence time course in response to a somatic holding voltage jump from -75 to +10 mV (magenta) is fitted by the kinetic of Na^+ entry and buffering (black line; *n* = 3; see Supplementary Methods below).

(b-c) The SBFI fluorescence time course near the AP initiation site (magenta) during five APs at 20 Hz (b) and 100 APs at 50 Hz (c) is fitted by the kinetics of Na^+ entry, buffering and removal (black). This gives the best-fit predictions for the kinetics of free intra-axonal Na^+ (orange; see Supplementary Methods below).

.

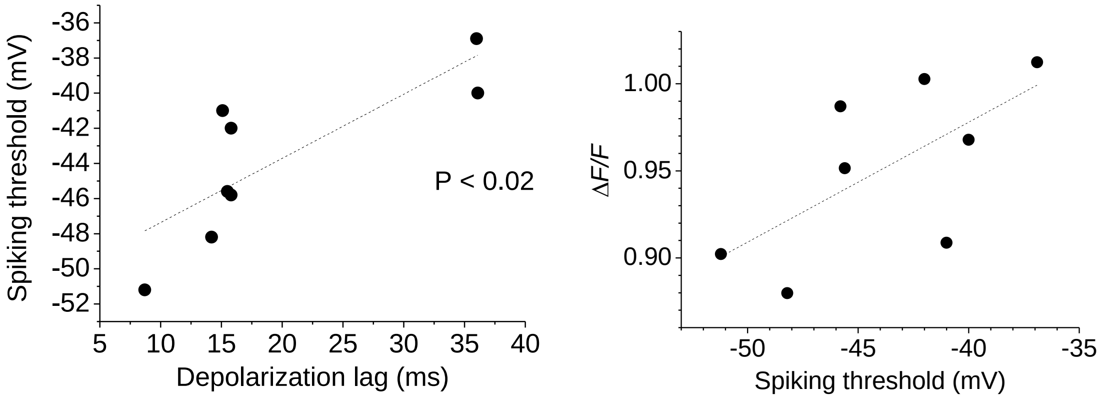

Supplementary Figure 5 The depolarization - spiking threshold relationship in Na^+ imaging experiments at 33°C: a one cell example.

The relationship between the rate of synaptic input-induced somatic depolarization, the cell spiking threshold, and axonal Na^+ entry signal (*ΔF/F*) in several recordings from one cell, as indicated (see Fig. 4g for further details).

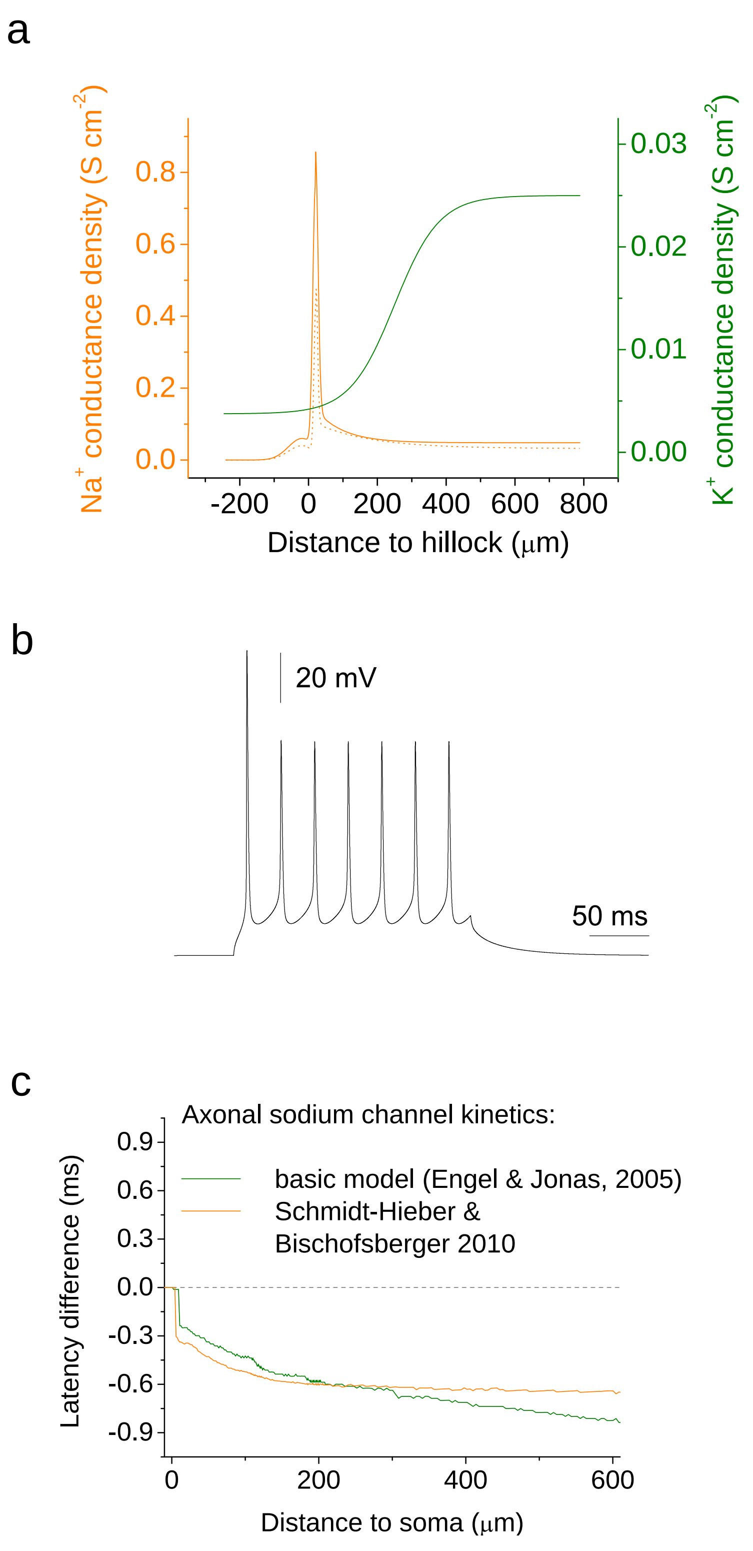

Supplementary Figure 6 The NEURON model of a dentate granule cell with distributed axonal conductance.

(a) For the sake of clarity, two ion conductances, representing Na^+ (orange) and K^+ (green) channels, were incorporated into the modeled granule cell axon [1, 2]. The conductance density was set as a function of distance *d* from the soma. An order-of-

magnitude increase in the sodium channel conductance (density) was set in the initial axonal segment to reflect a sharp rise in the expression of local Na^+ channels [3, 4]. The percentage of Na^+-channels that show slow inactivation [5] is given by 100% * (0.1 + 0.9 / (1 + $10^{0.2(d - 5)}$)). The distribution of Na^+ and K^+ conductance was adjusted empirically to allow cell firing patterns comparable with experimental observations (see b below); orange dotted line, distribution of the MF Na+ channel kinetics with the updated eight-stage channel activation model [4].

(b) A simulated pattern of somatic APs showing a marked spike amplitude adaptation in response to a 130 pA current injection. See Methods and Fig. 6 for further detail.

(c) Both measurement-based models of MF Na^+ channel kinetics published previously predict use-dependent broadening of the axonal spike initiation site. In our basic cell model (green; see Methods), axonal Na^+ channels inactivate approximately twice as fast as somatic ones [6] whereas a more recent model features faster kinetics of Na^+ channels [4] (orange; see dotted line in a for channel opening time course) .

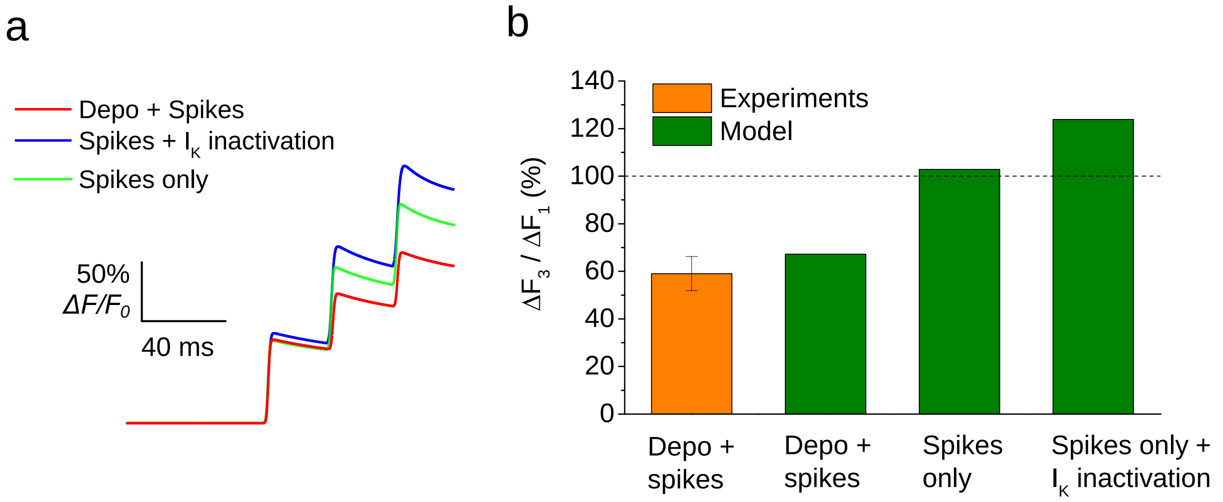

Supplementary Figure 7 Exploring the effect of Na^+ and K^+ channel inactivation on use-dependent Ca^{2+} influx near the axonal initial segment.

(a) Simulation examples of the Ca^{2+}-sensitive fluorescence (200 µM Fluo-4, see Methods for model description) in response to a train of APs, in conditions of spiking induced by somatic depolarization (current injection; red, Depo + Spikes), with spikes induced without depolarization (green, Spikes only), and with added inactivation of K+ channels (blue, + I_K inactivation), as indicated.

(b) Average $\Delta F_3/\Delta F_1$ ratios in Ca^{2+} imaging experiments (orange, see Fig. 5 for individual data points), and in the cell model in which spikes are induced by somatic depolarization (Depo + spikes), without depolarization (Spikes only), and with artificially enhanced depolarization-dependent inactivation of K^+ channels (Spikes only + I_K inactivation; inactivation was increased by the factor of five with respect to the kinetics used by Schmidt-Hieber and Bischofberger [4]; NEURON Model DB Accession 128079, Kin). Note that the latter condition leads to the effect opposite to that observed experimentally, thus suggesting a dominant role of Na^+, as opposed to K^+, channel inactivation in use-dependent Ca^{2+} entry.

SUPPLEMENTARY REFERENCES

1. Schmidt-Hieber, C., Jonas, P. & Bischofberger, J. Subthreshold dendritic signal processing and coincidence detection in dentate gyrus granule cells. *J Neurosci* 27, 8430-8441 (2007).
2. Scott, R., Ruiz, A., Henneberger, C., Kullmann, D.M. & Rusakov, D.A. Analog modulation of mossy fiber transmission is uncoupled from changes in presynaptic Ca2+. *J. Neurosci.* 28, 7765-7773 (2008).
3. Kress, G.J., Dowling, M.J., Meeks, J.P. & Mennerick, S. High threshold, proximal initiation, and slow conduction velocity of action potentials in dentate granule neuron mossy fibers. *J. Neurophysiol.* 100, 281-291 (2008).
4. Schmidt-Hieber, C. & Bischofberger, J. Fast sodium channel gating supports localized and efficient axonal action potential initiation. *J. Neurosci.* 30, 10233-10242 (2010).
5. Schmidt-Hieber, C., Jonas, P. & Bischofberger, J. Action potential initiation and propagation in hippocampal mossy fibre axons. *J Physiol* 586, 1849-1857 (2008).
6. Engel, D. & Jonas, P. Presynaptic action potential amplification by voltage-gated Na+ channels in hippocampal mossy fiber boutons. *Neuron* 45, 405-417 (2005).
